# Noninvasive Glioblastoma Testing: Multimodal Approach to Monitoring and Predicting Treatment Response

**DOI:** 10.1155/2018/2908609

**Published:** 2018-01-17

**Authors:** Maikel Verduin, Inge Compter, Danny Steijvers, Alida A. Postma, Daniëlle B. P. Eekers, Monique M. Anten, Linda Ackermans, Mark ter Laan, Ralph T. H. Leijenaar, Tineke van de Weijer, Vivianne C. G. Tjan-Heijnen, Ann Hoeben, Marc Vooijs

**Affiliations:** ^1^Department of Medical Oncology, School for Oncology and Developmental Biology (GROW), Maastricht University Medical Centre+, P.O. Box 5800, 6202 AZ Maastricht, Netherlands; ^2^Department of Radiotherapy (MAASTRO), School for Oncology and Developmental Biology (GROW), Maastricht University Medical Center+, Universiteitssingel 50, 6229 ER Maastricht, Netherlands; ^3^Department of Radiology and Nuclear Medicine, Maastricht University Medical Center+, P.O. Box 5800, 6202 AZ Maastricht, Netherlands; ^4^Proton Therapy Department South-East Netherlands (ZON-PTC), Maastricht University Medical Center+, P.O. Box 5800, 6202 AZ Maastricht, Netherlands; ^5^Department of Neurology, Maastricht University Medical Center+, P.O. Box 5800, 6202 AZ Maastricht, Netherlands; ^6^Department of Neurosurgery, Maastricht University Medical Center+, P.O. Box 5800, 6202 AZ Maastricht, Netherlands; ^7^Department of Neurosurgery, Radboud University Medical Center, 6500 HB Nijmegen, Netherlands; ^8^The-D-Lab: Decision Support for Precision Medicine, School for Oncology and Developmental Biology (GROW), Maastricht University Medical Centre+, Universiteitssingel 40, 6229 ER Maastricht, Netherlands

## Abstract

Glioblastoma is the most aggressive adult primary brain tumor which is incurable despite intensive multimodal treatment. Inter- and intratumoral heterogeneity poses one of the biggest barriers in the diagnosis and treatment of glioblastoma, causing differences in treatment response and outcome. Noninvasive prognostic and predictive tests are highly needed to complement the current armamentarium. Noninvasive testing of glioblastoma uses multiple techniques that can capture the heterogeneity of glioblastoma. This set of diagnostic approaches comprises advanced MRI techniques, nuclear imaging, liquid biopsy, and new integrated approaches including radiogenomics and radiomics. New treatment options such as agents targeted at driver oncogenes and immunotherapy are currently being developed, but benefit for glioblastoma patients still has to be demonstrated. Understanding and unraveling tumor heterogeneity and microenvironment can help to create a treatment regime that is patient-tailored to these specific tumor characteristics. Improved noninvasive tests are crucial to this success. This review discusses multiple diagnostic approaches and their effect on predicting and monitoring treatment response in glioblastoma.

## 1. Introduction

Glioblastoma (GBM) is the most aggressive primary brain tumor with an incidence of 2-3 cases per 100,000 people [1]. Currently, a median survival of approximately fourteen months is achieved with intensive multimodal treatment. However, despite this intensive treatment, there is no cure and recurrence of GBM is inevitable [2].

### 1.1. Diagnosis and Treatment

Diagnostic approaches in GBM are rapidly evolving. The diagnosis is currently based on the recently revised WHO criteria (2016) for the classification of central nervous system tumors. [3]. At present, histopathological investigation of a tissue sample from a suspected GBM lesion is the gold standard for the diagnosis. This is currently complemented by molecular diagnostics of which identification of O6-methylguanine-DNA-methyltransferase (*MGM*T) methylation, isocitrate dehydrogenase (*IDH*) mutation, and *1p19q* codeletion is currently the most valuable in daily clinical practice. Methylation of the *MGMT* gene is the only predictive marker for treatment response available and is predictive of an improved response to alkylating chemotherapy such as temozolomide [4, 5]. The *IDH* mutation status [6] and 1p19q codeletion [7] are of prognostic value but do not predict treatment response in patients with GBM. These markers are however of predictive value in oligodendroglioma [8, 9]. Additional markers such as telomerase reverse transcriptase promotor (*TERT*) mutations and alpha-thalassemia syndrome X-linked (*ATRX*) can already be used additionally in the classification of GBM subtypes [10, 11].

At the moment, the treatment schedule consists of neurosurgery, concurrent chemoradiation therapy, and adjuvant temozolomide. This provides a median progression-free survival of almost 7 months [2]. There is no standard treatment for the recurrent setting. Systemic treatment options include a temozolomide rechallenge, lomustine, and antiangiogenic therapy such as bevacizumab. However, effectiveness of these treatment options is limited. Additionally, reirradiation and re-resection can be considered depending on the localization of the tumor and condition of the patient [12].

### 1.2. Monitoring Treatment Response

Both during and after treatment for GBM, magnetic resonance imaging (MRI) is the main modality used in the follow-up and monitoring of treatment response. Difficulties arise in monitoring response when it comes to the differentiation between pseudoprogression, radiation necrosis, and actual tumor progression. Pseudoprogression is a physiologic posttreatment-related reaction of brain tissue, based on vascular and cellular injury from chemoradiation therapy. This leads to inflammation and increased permeability of the blood-brain barrier (BBB), causing an increase in contrast enhancement on MRI suggestive of tumor progression but without tumor recurrence [13, 14]. Radiation necrosis is a direct effect of radiation therapy, which can mimic tumor progression on imaging techniques but does not reflect actual progression of the tumor. Timing of MRI changes can help to differentiate between pseudoprogression, which occurs most commonly in the first three to six-month posttreatment, and radiation necrosis which occurs six months to several years after treatment [15].

Markers are also needed for monitoring treatment response for patients treated with immunotherapy. On MRI, these patients may first show an increase in size or even the formation of new (pseudo) lesions due to the antitumor-mediated immune response and localized inflammation, which does not necessarily define progressive disease [16, 17]. At the moment, differentiating pseudoprogression from actual tumor progression remains difficult and currently only follow-up imaging with conventional imaging methods is available to define this. Therefore, new imaging techniques and/or biomarkers to further characterize the origin of the imaging changes that are observed are needed to overcome these challenges.

On the other hand, another phenomenon on MRI called pseudoresponse can also occur, mainly during treatment with vascular endothelial growth factor (*VEGF*) signaling pathway modifying agents such as bevacizumab. Bevacizumab induces a steroid-like effect by normalizing the permeability of the BBB, leading to a rapid decrease in contrast enhancement. Thus, while the imaging reflects reduced contrast and suggests a posttreatment response, the effects on overall survival are minimal [13, 18, 19].

### 1.3. Tumor Heterogeneity

Tumor heterogeneity poses one of the most important challenges in the current diagnosis and treatment of GBM, and it is one of the main difficulties when it comes to finding new treatment options. GBM shows varying tumor characteristics both between patients and within individual tumors [20, 21]. Current histological analysis cannot capture the full spectrum of genotypic and phenotypic characteristics, especially when only a single biopsy can be taken.

The intratumoral heterogeneity poses a great challenge in predicting sensitivity and resistance to systemic therapies. As one clone within the tumor may be sensitive to one form of treatment, another clone might harbor certain resistance mechanisms to this treatment in its specific tumor microenvironment. Intratumoral heterogeneity is a dynamic process which changes over time and during treatment which poses challenges in the recurrent setting of GBM, as research has shown recurrent tumors usually show resistance to the traditional treatment options and expresses different mutations when compared to the original tumor [20, 22].

Since conventional MRI cannot reflect tumor heterogeneity, before this can be used as a parameter in daily clinical practice, improved diagnostic approaches should be developed to identify this heterogeneity.

### 1.4. Noninvasive Glioblastoma Testing

Noninvasive glioblastoma testing (NIGT) combines noninvasive (i.e., nonsurgical) techniques to represent the tumor as a whole and provides information on driver mechanisms and tumor microenvironment, all of which are factors that can/should be incorporated into the treatment regime. This can be especially helpful in the selection of patients in order to better predict response to new therapeutic targets, as the current options available are limited. Also, the brain is less easily accessible for taking repeated biopsies, which stresses the need for noninvasive approaches. New integrated approaches such as radiogenomics and radiomics can also be an important part of NIGT. Radiogenomics is an experimental diagnostic and predictive tool which studies the association between (qualitative) imaging features and molecular markers [23]. Radiomics on the other hand uses a computational analysis to extract quantifiable information about the underlying tumor characteristics by high-throughput mining of large amounts of quantitative features from images based on, for example, textures, intensities, and shapes [24, 25]. Radiomics has already been more extensively studied in, for example, head-and-neck cancer [26, 27] and lung cancer [28].

The objective of this review is to discuss the already established approaches as well as future diagnostics used for monitoring and prediction of treatment response in patients with GBM by creating a so-called NIGT platform. This includes a multimodal approach to fully capture the complexity and heterogeneity of GBM with the use of conventional techniques such as imaging techniques, enhanced by computational approaches, and the use of circulating biomarkers.

## 2. Magnetic Resonance Imaging

MRI is clinically used in diagnosis and follow-up of cerebral tumors. The use of imaging to predict patient survival has been applied since as early as 1996 [29, 30]. Features found to be correlating with a longer survival in GBM are the presence of nonenhancing tumor and the absence of either edema, satellites, or multifocality [31]. The Visually Accessible Rembrandt Images (VASARI) research project aimed to make MRI features more accurate and reproducible. In this project, a set of 24 observations describing the morphology of brain tumors on contrast-enhanced MRI were reported and analyzed for their prognostic significance on overall survival [32, 33].

### 2.1. Monitoring Treatment Response

For the evaluation of tumor response after first-line treatment, the Response Assessment in Neuro-Oncology (RANO) criteria of 2010 is currently used [34]. A major drawback of these criteria is its nonvolumetric criteria and lack of use of advanced MR techniques. For instance, with using only these RANO criteria, pseudoprogression cannot be distinguished from radiation necrosis or disease recurrence [35].

Several advanced MR techniques have been developed to improve standard contrast-enhanced MRI, such as diffusion-weighted imaging (DWI), perfusion imaging, and magnetic resonance spectroscopy (MRS) [15]. DWI displays the cellularity within tissue by detecting free diffusion of water molecules. The apparent diffusion coefficient (ADC) is a derived DWI parameter in which the T2 signal from the original DWI is excluded to overcome the so-called “T2 shine-through effect,” which causes a high signal on DWI that is not due to restricted diffusion. DWI and ADC are widely used in tumor imaging, where a decrease in ADC signal has been shown to correlate with increasing tumor cellularity while an increase in signal correlates with decreasing cellularity as a result of successful treatment [36, 37].

A relatively new DWI technique that has been developed is functional diffusion map (fDM) imaging, which reflects differences in ADC signal over time. This fDM analysis has shown to be able to distinguish progressive tumors from stable and partially responsive tumors [38, 39]. Although this technique is promising, there is a great variability in protocols collecting and processing DWI/ADC data between different vendors, standing in the way of wide-scale use [40].

Perfusion images can be acquired in various ways, with dynamic susceptibility-weighted contrast-enhanced MR (DSC MR) being most widely used. Other perfusion techniques include dynamic-contrast-enhanced MR (DCE MR), which is comparable to DSC MR, and arterial spin labeling (ASL) perfusion, which does not require intravenous contrast but is more susceptible for artifacts. DSC MR is able to assess cerebral microvasculature by following an administered contrast agent as it passed through the microvasculature. Tumors tend to have a higher number and larger volumes of blood vessels. Furthermore, remodeling of the extracellular matrix disturbs the BBB and causes leakage of contrast [14, 41, 42]. By comparing, for example, tumor areas with healthy brain tissue, relative cerebral blood volumes (rCBV) can be measured [14, 41]. The presence of high rCBV has been shown to represent active neovascularization and viable tumor, whereas normal rCBV in apparent lesion progression could point to, for example, chemoradiation effects and thereby exclude pseudoprogression and radionecrosis [43, 44].

MRS can be used to measure the distribution of chemical metabolites in brain tissue and thereby identifying differences in metabolic turnover of brain tissue. As high-grade tumors are highly metabolically active and are accompanied by a leaky blood-brain barrier, regional differences can be found in the spectroscopic profile in tumor depositions, compared to necrosis, pseudoprogression, and healthy brain tissue. In ^1^H-spectroscopy, elevated peaks of lipid, lactate, choline, and myoinositol and reduced NAA signal are typical findings in primary brain tumors [45, 46]. Due to patient and tumor-specific differences, an unequivocal threshold of metabolic signal ratios cannot be determined making it difficult to establish uniform guidelines and accuracy; however, MRS changes in time can be of help to strengthen suspicions on, for example, tumor progression or response [47]. MRS alone therefore has a moderate diagnostic performance in differentiating glioma recurrence from radiation necrosis and should always be combined with other advanced imaging technologies [48].

### 2.2. Tumor Heterogeneity and Predicting Treatment Response

GBM is subdivided into four subcategories based on histopathological features and specific mutations and molecular markers: proneural, neural, mesenchymal, and classical subtypes. Each subset is associated with specific mutations; therefore, identification of the subtype by radiogenomics can provide information on driver mechanisms in the tumor. Between these subtypes, the proneural subtype is thought to have the most favorable prognosis [49]. Also, different subtypes react differently to different treatment options [50]. Radiogenomics can be applied to predict the GBM subtype. Volumes of both contrast enhancement and necrosis are higher in tumors with the mesenchymal subtype compared to the proneural subtype. GBMs with less than 5% tumor enhancement are mostly of the proneural subtype. On the other hand, GBMs with less than 5% nonenhanced tumor rarely represent proneural tumors and are more linked to the classical or mesenchymal subtype [51, 52].

Radiogenomics can also be used as a tool to predict mutation status. *IDH1*-mutation status is associated with a localization of the tumor in the frontal lobe, a higher percentage of noncontrast-enhancing part of the tumor, and the presence of cysts on MRI [53, 54]. MRS has recently been used to predict *IDH* mutation status. MRS can measure elevated levels of 2-HG metabolite which is a surrogate marker for *IDH*-mutated tumor cells and can correctly identify *IDH* mutation status in 88.6% of patients (sensitivity 89.5%, specificity 81.3%). However, further technical improvement of this technique, including voxel localization, as well as understanding of the impact of tumor heterogeneity on MRS is needed before it can be used in daily clinical practice [55, 56].

*MGMT*-methylated tumors tend to be lateralized to the left temporal lobe whereas *MGMT*-unmethylated tumors are more frequent in the right hemisphere. This may be due to asymmetry in brain structure, function, and gene expression between the hemispheres [57]. *MGMT*-unmethylated tumors have a higher percentage of tumor enhancement and T2/FLAIR hyperintensity when compared to *MGMT*-methylated tumors [53]. Several imaging features are potential indicative of *MGMT* methylation such as mixed nodular enhancement, limited edema, and moderately increased rCBV [23].

The presence of the *1p19q* codeletion is linked to classical oligodendroglial MRI characteristics such as heterogeneous T2 signal intensity and the presence of calcifications. Advanced imaging techniques have not yet shown to improve the capacity to identify the *1p19q* codeletion over conventional MRI to identify oligodendroglial tumors [23].

Epidermal growth factor receptor (*EGFR*) amplification is associated with a significant higher percentage of contrast enhancement and T2/FLAIR hyperintensity compared with tumors lacking *EGFR* amplification. Also, *EGFR* amplification and *EGFRvIII* mutant GBMs are commonly associated with localization in the left temporal lobe [53].

Apart from already mentioned molecular markers, other known driver genes, such as phosphatase and tensin homolog (*PTEN*), platelet-derived growth factor receptor A (*PDGFRA*), cyclin-dependent kinase inhibitor 2A (*CDKN2A*), retinoblastoma 1 (*RB1*), and tumor protein 53 (*TP53*) are also under investigation and significant image correlations for these genes have already been identified [58].

The aforementioned advanced imaging techniques can also aid in exploring tumor heterogeneity. Both CBV and ADC measurements are found to be influenced by tumor aggressiveness, and it is suggested that the heterogeneous genetic and cellular expression patterns within GBM influence anatomic and physiologic MR imaging [59]. These techniques can also guide neurosurgeons in determining the biopsy location.

MRI-based radiomics for GBM is a relatively new area for which little research has been published to this date. A study of 82 GBM patients reported favorable results in the performance of texture features in predicting the molecular subtype and 12-month survival [60]. For the prediction of 12-month survival based on pattern analysis, sensitivity and specificity of 0.86 and 0.64, respectively, are reported. The prediction of GBM subtype was also investigated. Accuracy for classical, mesenchymal, neural, and proneural subtypes was 0.88, 0.70, 0.85, and 0.93, respectively [61]. Another study used machine-learning techniques and found an accuracy of almost 80% in predicting overall survival and an accuracy of 76% in predicting the molecular subtype [62].

MRI texture analysis has been found to be able to facilitate in characterizing intratumoral heterogeneity and may therefore aid in identifying genetically different components of the tumor and understanding its consequences for prognosis, treatment sensitivity, and resistance [58]. It has been shown that radiomics features are able to visualize spatial gene expression within a tumor [63]. Patients can be subdivided into different clusters using texture features. For example, one study divided patients into the “premultifocal,” “spherical,” or “rim-enhancing” cluster based on quantitative imaging features. Each of these clusters was linked to different signaling pathways and microarrays and has been shown to be prognostic for survival [64].

Radiomics was found to have prognostic value for both survival and progression in patients with recurrent GBM receiving bevacizumab. Therefore, it might be possible to develop pretreatment biomarkers based on radiomics to predict benefit from bevacizumab [65, 66]. These findings illustrate the possibilities of applying radiomics in the prediction of treatment response in patients with GBM. Further optimization of this technique and validation of radiomics profiles as predictors for different mutation statuses and/or survival are needed before it can be used in clinical practice.

## 3. Nuclear Imaging

Molecular imaging by the use of positron emission tomography (PET) is increasingly being implemented into clinical practice for treatment planning and response monitoring of GBM. The most common is fluorodeoxyglucose (^18^F-FDG) PET imaging; however, compared to other organ systems, ^18^F-FDG-PET imaging of brain tumors presents unique challenges because of the high background glucose metabolism of normal gray matter masking detection of malignant lesions. Thus, the use of ^18^F-FDG-PET in brain tumors albeit is limited, although in high-grade glioma, ^18^F-FDG-PET imaging can be used to identify metabolically active disease which correlates with tumor grade [67, 68]. Because of the limited utility of ^18^F-FDG-PET, the RANO working group has recommended the use of radio-labeled amino acid tracers for PET (AA-PET) instead [69].

Amino acid tracers such as O-(2-[^18^F]-fluoroethyl)-L-tyrosine (^18^F-FET) and L-[methyl-^11^C]methionine (^11^C-MET) are currently applied in clinical practice in GBM. Both ^18^F-FET and ^11^C-MET show rapid uptake into tumors and can be visualized with high contrast [70]. ^11^C has a short half-life time of 20 minutes, making it less useful for clinical practice. ^18^F has a much longer half-life time (120 minutes) and is only taken up by cells through specific L-transporters—LAT2—that are highly and predominantly expressed on glioma tumor cells. This ensures a high and selective uptake of the tracer in tumor tissue, with a low to negligible background signal in normal brain tissue or in surrounding inflammatory areas. Thus, ^18^F-FET-PET has a high sensitivity and specificity for the detection of malignant gliomas [71–74]. A biopsy-controlled study has shown that with a combination of MRI and ^18^F-FET-PET, a sensitivity of 93% and a specificity of 94% can be achieved [71]. Other amino acid tracers currently under investigation include ^18^F-FDOPA (phenyl alanine) PET, a dopaminergic tracer, and alpha^11^C-L-methyl-tryptophan PET, a tryptophan analog.

### 3.1. Monitoring Treatment Response

^18^F-FET-PET currently has multiple potential clinical applications including the monitoring of treatment response and can distinguish tumor recurrence from radiation necrosis or pseudoprogression [69]. A study investigating ^18^F-FET for PET-guided radiotherapy concluded that size and geometrical location of gross tumor volume and biological tumor volume, defined by ^18^F-FET uptake, were significantly different in patients where the biological tumor volume extended up to 10–20 mm from the margin of contrast enhancement on MRI, potentially improving local tumor control due to improved radiotherapy planning using ^18^F-FET-PET [75]. Multiple studies have shown that both ^18^F-FET-PET and ^18^F-FDOPA-PET have a higher diagnostic accuracy than conventional MRI in differentiating glioma recurrence from posttreatment tissue changes. For example, studies show a sensitivity and specificity of 92.3% and 44.4%, respectively, for MRI compared to 100% and 88.89% for ^18^F-FDOPA-PET [76–79]. A prospective study investigating the predictive value of ^18^F-FET-PET in patients treated with chemoradiation has shown that a decrease of ^18^F-FET-PET accumulation reflects tumor response to the therapeutic intervention at an early stage of the disease and predicts outcome, whereas contrast-enhanced MRI did not [78].

### 3.2. Tumor Heterogeneity and Predicting Treatment Response

Correlation between different types of AA-PET standard uptake values (SUV) and molecular markers, in the context of radiogenomics, is currently under investigation. A longitudinal prospective study has investigated ^18^F-FET-PET as an imaging biomarker, and they concluded that the biological tumor volume before treatment was a strong prognostic marker for both overall- and progression-free survival independent of treatment as well as *MGMT* promoter methylation and other patient- and tumor-related factors. Moreover, tumor uptake kinetics before and after treatment (i.e., TAC curves) is correlated with progression-free survival [80].

A recent study demonstrated the relationship between ^11^C-MET-PET and *IDH1* mutation and found that SUVmax and SUVratio were inversely correlated with *IDH1* mutation [81]. Moreover, a study which combined MRI and alpha[C-11]-L-methyl-tryptophan PET imaging showed prognostic imaging factors such as T1-contrast/PET volume ratios and metabolic volume, which are associated with *EGFR* amplification and *MGMT* methylation status [82]. To assess the potential of radiogenomics as a diagnostic and predictive tool, well-defined preclinical models with specific driver mutations are needed that can be used to validate the sensitivity and specificity of radiogenomics. The ability to identify prognostic or molecular response markers based on imaging features derived from routine diagnostic procedures (MRI, PET, and computed tomography (CT)) provides an attractive way of predicting treatment response in GBM.

Tumor hypoxia is a common feature of the tumor microenvironment in GBM and contributes to increased malignancy, poor prognosis, and resistance to radiotherapy and alkylating chemotherapy such a temozolomide [83–85]. Acute and chronic hypoxic areas fluctuate in human tumors and contribute to spatial and temporal intratumoral heterogeneity [86, 87]. This has a significant impact on resistance to conventional treatment. Therefore, GBM patients might benefit from hypoxia-targeting drugs [21]. Molecular imaging of tumor hypoxia could aid in the selection of patients with hypoxic tumors, which could benefit from specific antihypoxic therapies. The efficacy of antihypoxic treatments will depend on the presence of hypoxia. Several 2-nitroimidazoles, labeled with ^18^F, have already been investigated in patients to identify hypoxia [88]. In extensive preclinical models and clinical trials, ^18^F-HX4-PET has shown to be a promising and nontoxic probe for hypoxia [89–91]. Repeated hypoxia imaging during the course of disease and treatment will demonstrate the extent of spatial and temporal fluctuations in tumor hypoxia and is likely important in scheduling hypoxia-modifying drug in combination with conventional treatments.

## 4. Liquid Biopsy

Liquid biopsy (LB) has entered clinical practice in the treatment of several cancer types, including breast and colorectal cancer [92, 93]. LB studies circulating biomarkers which refer to measurable biological molecules found in blood, urine, and or other body fluids, like cerebrospinal fluid (CSF). Although LB has refined the individual treatment for several cancer subtypes, relatively little progress has been made with regard to validation of circulating biomarkers for primary brain tumors. Nevertheless, although the translation of biomarker development into neuro-oncology is lagging behind compared to general oncology, the prerequisites for adequate extrapolation are present, mostly in experimental studies [94]. In these studies, circulating tumor cells (CTCs), circulating free nuclear acids (cfNA), extracellular vesicles, and circulating proteins and metabolites have been described.

For brain tumors, where noninvasive procedures are complex, precarious, and may be nonrepresentative for outcome, circulating biomarkers pose a realistic option. For the inoperable patients, which mostly occurs in the recurrent setting, circulating biomarkers could be the source of a molecular profile of the relapsed tumor, allowing clinicians to identify potentially druggable molecular alterations driving recurrence.

### 4.1. Monitoring Treatment Response

miRNAs are small (about 21–24 nucleotides) noncoding regulatory RNA molecules and can be detected as cell-free entities or as the content of circulating extracellular vesicles (EVs) in plasma/serum or CSF. EVs are small nanometer size membrane-enclosed particles that are released from GBM living tumor cells. EVs that can be isolated from both blood and CSF are a rich source of tumor-derived molecules such as DNA, miRNA, mRNA, proteins, lipids, and metabolites, because the structure of EVs protects them from nucleases and proteases [95].

The human genome encodes for miRNAs which have been shown to regulate most hallmarks of tumor development and progression via transcriptional silencing or translation inhibition of both oncogene and suppressor genes and have tumor/tissue-specific signatures [96]. miRNAs have been described in GBM, mostly from resected specimens. miR-21 is the most reliable plasma biomarker in glioma diagnosis and seems to be valuable distinguishing tumor progression from pseudoprogression or radionecrosis [97]. Exosomal miRNA-21 in CSF of glioma patients has been shown to correlate with glioma recurrence [98]. An increase in levels of blood-born annexin V-positive microvesicles during chemoradiation is associated with earlier recurrence and shorter overall survival. Since the number of patients included in this analysis was only small (*n* = 16), further investigation is needed [99].

### 4.2. Tumor Heterogeneity and Predicting Treatment Response

The ability to detect CTCs in GBM patients has been established [100]. Also, using single-cell genome sequencing, some unique mutations were found in the CTCs as well as in the parental tumor [101]. However, the most important limiting factor for the clinical implementation of CTCs is their scarcity, which makes it difficult to adequately assess GBM heterogeneity. Also, it has not yet been established in GBM if CTCs can be identified which show characteristics of brain tumor-initiating cells.

Circulating tumor DNA (ctDNA) is much more abundant than CTCs and contains the mutations present in tumors [102]. In GBM patients, a lower rate of ctDNA is detected compared to other solid tumors, mainly due to the only partially disrupted BBB. In several small retrospective studies, ctDNAs were successfully detected in GBM patients and multiple molecular alterations were characterized including loss of heterozygosity (LOH) in chromosome arms *1p*, *19q* and *10q*, *IDH1*, and *EGFRvIII* mutations as well as methylation of promoters of *MGMT*, *PTEN*, and *CDKN2A* [103–105]. Only few studies have reported the plasma half-life of ctDNA. The available data propose that the fast turnover of ctDNA reflects tumor homeostasis [106]. Until now, the clinical utility of candidate ctDNAs as biomarkers for patients with GBM has not been demonstrated and large-scale prospective studies are needed before their implementation in clinical practice.

miR-130a was found to positively correlate with temozolomide response in GBM patients, independent from *MGMT* methylation status [107]. miR-603 is another regulator of *MGMT* and could complement assessment of *MGMT* methylation, which alone cannot completely explain temozolomide efficiency, as a predictive marker for treatment response [108]. miR-181d levels in the serum of GBM patients are also shown to correlate with response to temozolomide, since this miRNA, like miR-603, is directly involved in the downregulation of *MGMT* [109].

Mutant *IDH* enzymes acquire neomorphic enzymatic activity, thereby catalyzing the production of D-2-hydroxyglutarate (D2HG), an oncometabolite that accumulates at high levels and inhibits several enzymes notably involved in histones and DNA demethylation [110]. In most patients with *IDH1*/2 mutant gliomas, plasma D2HG values are in the normal range [111], suggesting limited clinical value of this oncometabolite. However, combining this technique with MRS which can, as mentioned before, measure D2HG metabolite concentrations in brain tissue, might be useful in exploring *IDH* mutation status.

Several circulating proteins have been evaluated and include proteins with cell lineage such as *GFAP* [112], *NCAM* [113], and *S100B* [114], matricellular proteins and matrix metalloproteinases such as *YKL*-40, *MMP2*, *MMP9* [115], *TIMP*-1, and *osteopontin* [116], and cytokines [117], growth factors, and growth factor receptors such as *VEGF*, *FGF-2*, *PlGF*, *IGFBP-5*, *EGFR*, *VEGFR1* [118], and *TGF-β1* [119]. Further validation of these biomarkers is warranted.

## 5. Discussion and Future Perspectives

Advanced MR imaging techniques, nuclear imaging, liquid biopsy and integrated radiogenomics, and radiomics approaches are examples of noninvasive diagnostic methods to uncover underlying tumor characteristics. GBM is a challenging tumor both from a diagnostic and therapeutic point of view. Recent advances are made when it comes to molecular markers and the understanding of underlying driver oncogenes and tumor microenvironment, all factors which contribute to treatment sensitivity and resistance. Diagnostic methods to accurately identify these factors and their impact on outcome are needed to be able to put this knowledge to clinical use.

Tumor heterogeneity poses a big challenge in the use of targeted treatment approaches. Apart from heterogeneity on the genetic level, nongenetic factors such as the tumor microenvironment also influence the development on cancer cell populations [120]. Different niches have been identified in GBM harboring very different epigenetic and environmental factors which also play a role in treatment resistance and heterogeneity [121]. These differences make it challenging to create one uniform treatment schedule for GBM, and a comprehensive insight into the behavior of these distinct tumor cell populations is needed.

The diagnostic modalities previously discussed all have future possibilities to improve the understanding of tumor heterogeneity and the prediction of treatment response as well as the monitoring of treatment response with regard to the differentiation of pseudoprogression, radiation necrosis, and actual tumor progression. The latter is especially important when it comes to patients treated with immunotherapy, for which no adequate distinction between pseudoprogression and actual progression can currently be made.

MRI is already well established within the clinic of GBM; further optimization through higher resolutions (e.g., ultrahigh field MRI), wider use of advanced imaging techniques, and further research, including clinical validation, on the application of radiogenomics will improve the diagnostic power of MRI. The same applies to nuclear imaging, especially different types of AA-PET for which additional studies on known amino acid tracers and the development of new tracers to improve diagnostic accuracy, both in the setting of the primary diagnosis as well as in the monitoring of treatment response and patient follow-up, are warranted. Radiomics is an important topic in different types of solid tumors and still relatively new in GBM. Radiomics analysis is one of the most promising techniques for the differentiation between different GBM subsets and in evaluating and monitoring intratumoral heterogeneity [122]. Ideally predictive radiomics models are created as predictors for GBM subtypes as has already been established using radiomics in other types of solid tumors [24]. Before radiomics can be applied, prospective validation is needed as well as standardization of imaging protocols, imaging segmentation, and feature extraction to ensure interoperability of multicenter radiomics studies [123].

Liquid biopsy provides a different approach for understanding tumor characteristics. Further research should focus on determining the clinical value of liquid biopsy and also which liquid source and which biomarker technique to use. Also, the possibility to use liquid biopsy in patients after a tumor resection as a marker for tumor recurrence has yet to be studied. Tumor heterogeneity will remain an important pitfall in liquid biopsy technique; this might be overcome by combining liquid biopsy with other noninvasive markers but will remain a challenge.

Future research should focus on determining the sensitivity and specificity and validating the techniques previously discussed for GBM. Being able to understand and unravel intratumoral heterogeneity provides clinicians with important information to create the most optimal treatment regime, and this should therefore be the focus of future studies.

NIGT offers a noninvasive panel to understand the driver mechanisms as potential treatment targets as well as identifying the tumor microenvironment. Combining different diagnostic modalities aims to achieve optimal diagnostic power for identification of tumor characteristics. Understanding the tumor microenvironment (e.g., hypoxia, angiogenesis, and immune infiltration) can help in finding new ways to treat GBM or to alter the tumor microenvironment to improve the effectiveness of systemic therapies and radiotherapy. Unique to the brain microenvironment is the BBB which limits effectiveness of therapeutic agents. Advances in imaging allow the visualization of changes in the tumor microenvironment and tissue architecture as a response to treatment and can therefore serve as a marker for treatment response.

Given the limitations of each of the currently available noninvasive tools, these diagnostic methods are ideally combined into a so-called NIGT platform: a multimodal noninvasive approach to visualize the tumor and its underlying tumor characteristics in a spatially and temporally relevant manner. Using this NIGT platform, predictive models for GBM can be created, both in the primary and the recurrent setting. This will guide clinicians in selecting the appropriate treatment option, treatment monitoring, and adaptation in the era of patient-tailored precision medicine.

## References

[1] Urbanska K., Sokolowska J., Szmidt M., Sysa P. (2014). Glioblastoma multiforme - an overview. *Contemporary oncology*.

[2] Stupp R., Mason W. P., van den Bent M. J. (2005). Radiotherapy plus concomitant and adjuvant temozolomide for glioblastoma. *The New England Journal of Medicine*.

[3] Louis D. N., Perry A., Reifenberger G. (2016). The 2016 World Health Organization classification of tumors of the central nervous system: a summary. *Acta Neuropathologica*.

[4] Esteller M., Garcia-Foncillas J., Andion E. (2000). Inactivation of the DNA-repair gene *MGMT* and the clinical response of gliomas to alkylating agents. *The New England Journal of Medicine*.

[5] Hegi M. E., Diserens A. C., Gorlia T. (2005). *MGMT* gene silencing and benefit from temozolomide in glioblastoma. *The New England Journal of Medicine*.

[6] Dahlrot R. H., Kristensen B. W., Hjelmborg J., Herrstedt J., Hansen S. (2013). A population-based study of high-grade gliomas and mutated isocitrate dehydrogenase 1. *International Journal of Clinical & Experimental Pathology*.

[7] Aldape K., Burger P. C., Perry A. (2007). Clinicopathologic aspects of 1p/19q loss and the diagnosis of oligodendroglioma. *Archives of Pathology & Laboratory Medicine*.

[8] Cairncross G., Wang M., Shaw E. (2013). Phase III trial of chemoradiotherapy for anaplastic oligodendroglioma: long-term results of RTOG 9402. *Journal of Clinical Oncology*.

[9] Cairncross J. G., Wang M., Jenkins R. B. (2014). Benefit from procarbazine, lomustine, and vincristine in oligodendroglial tumors is associated with mutation of *IDH*. *Journal of Clinical Oncology*.

[10] Eckel-Passow J. E., Lachance D. H., Molinaro A. M. (2015). Glioma groups based on 1p/19q, *IDH*, and TERT promoter mutations in tumors. *The New England Journal of Medicine*.

[11] van den Bent M. J., Weller M., Wen P. Y., Kros J. M., Aldape K., Chang S. (2017). A clinical perspective on the 2016 WHO brain tumor classification and routine molecular diagnostics. *Neuro-Oncology*.

[12] van Linde M. E., Brahm C. G., de Witt Hamer P. C. (2017). Treatment outcome of patients with recurrent glioblastoma multiforme: a retrospective multicenter analysis. *Journal of Neuro-Oncology*.

[13] Brandsma D., van den Bent M. J. (2009). Pseudoprogression and pseudoresponse in the treatment of gliomas. *Current Opinion in Neurology*.

[14] Korfiatis P., Erickson B. (2014). The basics of diffusion and perfusion imaging in brain tumors. *Applied Radiology*.

[15] Ellingson B. M., Chung C., Pope W. B., Boxerman J. L., Kaufmann T. J. (2017). Pseudoprogression, radionecrosis, inflammation or true tumor progression? Challenges associated with glioblastoma response assessment in an evolving therapeutic landscape. *Journal of Neuro-Oncology*.

[16] Huang R. Y., Neagu M. R., Reardon D. A., Wen P. Y. (2015). Pitfalls in the neuroimaging of glioblastoma in the era of antiangiogenic and immuno/targeted therapy - detecting illusive disease, defining response. *Frontiers in Neurology*.

[17] Okada H., Weller M., Huang R. (2015). Immunotherapy response assessment in neuro-oncology: a report of the RANO working group. *The Lancet Oncology*.

[18] Chang J. H., Kim C. Y., Choi B. S., Kim Y. J., Kim J. S., Kim I. A. (2014). Pseudoprogression and pseudoresponse in the management of high-grade glioma: optimal decision timing according to the response assessment of the neuro-oncology working group. *Journal of Korean Neurosurgical Society*.

[19] Batchelor T. T., Sorensen A. G., di Tomaso E. (2007). AZD2171, a pan-VEGF receptor tyrosine kinase inhibitor, normalizes tumor vasculature and alleviates edema in glioblastoma patients. *Cancer Cell*.

[20] Qazi M. A., Vora P., Venugopal C. (2017). Intratumoral heterogeneity: pathways to treatment resistance and relapse in human glioblastoma. *Annals of Oncology*.

[21] Vartanian A., Singh S. K., Agnihotri S. (2014). GBM’s multifaceted landscape: highlighting regional and microenvironmental heterogeneity. *Neuro-Oncology*.

[22] Johnson B. E., Mazor T., Hong C. (2014). Mutational analysis reveals the origin and therapy-driven evolution of recurrent glioma. *Science*.

[23] Smits M., van den Bent M. J. (2017). Imaging correlates of adult glioma genotypes. *Radiology*.

[24] Aerts H. J., Velazquez E. R., Leijenaar R. T. (2014). Decoding tumour phenotype by noninvasive imaging using a quantitative radiomics approach. *Nature Communications*.

[25] Lambin P., Rios-Velazquez E., Leijenaar R. (2012). Radiomics: extracting more information from medical images using advanced feature analysis. *European Journal of Cancer*.

[26] Leijenaar R. T., Carvalho S., Hoebers F. J. (2015). External validation of a prognostic CT-based radiomic signature in oropharyngeal squamous cell carcinoma. *Acta Oncologica*.

[27] Parmar C., Grossmann P., Rietveld D., Rietbergen M. M., Lambin P., Aerts H. J. W. L. (2015). Radiomic machine-learning classifiers for prognostic biomarkers of head and neck cancer. *Frontiers in Oncology*.

[28] Wu W., Parmar C., Grossmann P. (2016). Exploratory study to identify radiomics classifiers for lung cancer histology. *Frontiers in Oncology*.

[29] Hammoud M. A., Sawaya R., Shi W., Thall P. F., Leeds N. E. (1996). Prognostic significance of preoperative MRI scans in glioblastoma multiforme. *Journal of Neuro-Oncology*.

[30] Pierallini A., Bonamini M., Pantano P. (1998). Radiological assessment of necrosis in glioblastoma: variability and prognostic value. *Neuroradiology*.

[31] Pope W. B., Sayre J., Perlina A., Villablanca J. P., Mischel P. S., Cloughesy T. F. (2005). MR imaging correlates of survival in patients with high-grade gliomas. *American Journal of Neuroradiology*.

[32] Wangaryattawanich P., Hatami M., Wang J. (2015). Multicenter imaging outcomes study of The Cancer Genome Atlas glioblastoma patient cohort: imaging predictors of overall and progression-free survival. *Neuro-Oncology*.

[33] (2013). The Cancer Imaging Archive. *Wiki for the VASARI feature set*.

[34] Wen P. Y., Macdonald D. R., Reardon D. A. (2010). Updated response assessment criteria for high-grade gliomas: response assessment in neuro-oncology working group. *Journal of Clinical Oncology*.

[35] Wen P. Y., Chang S. M., Van den Bent M. J., Vogelbaum M. A., Macdonald D. R., Lee E. Q. (2017). Response assessment in neuro-oncology clinical trials. *Journal of Clinical Oncology*.

[36] Chenevert T. L., McKeever P. E., Ross B. D. (1997). Monitoring early response of experimental brain tumors to therapy using diffusion magnetic resonance imaging. *Clinical Cancer Research*.

[37] Sugahara T., Korogi Y., Kochi M. (1999). Usefulness of diffusion-weighted MRI with echo-planar technique in the evaluation of cellularity in gliomas. *Journal of Magnetic Resonance Imaging*.

[38] Moffat B. A., Chenevert T. L., Lawrence T. S. (2005). Functional diffusion map: a noninvasive MRI biomarker for early stratification of clinical brain tumor response. *Proceedings of the National Academy of Sciences of the United States of America*.

[39] Moffat B. A., Chenevert T. L., Meyer C. R. (2006). The functional diffusion map: an imaging biomarker for the early prediction of cancer treatment outcome. *Neoplasia*.

[40] Schmainda K. M. (2012). Diffusion-weighted MRI as a biomarker for treatment response in glioma. *CNS Oncology*.

[41] Gahramanov S., Raslan A. M., Muldoon L. L. (2011). Potential for differentiation of pseudoprogression from true tumor progression with dynamic susceptibility-weighted contrast-enhanced magnetic resonance imaging using ferumoxytol vs. gadoteridol: a pilot study. *International Journal of Radiation Oncology, Biology, Physics*.

[42] Nasseri M., Gahramanov S., Netto J. P. (2014). Evaluation of pseudoprogression in patients with glioblastoma multiforme using dynamic magnetic resonance imaging with ferumoxytol calls RANO criteria into question. *Neuro-Oncology*.

[43] Law M., Young R. J., Babb J. S. (2008). Gliomas: predicting time to progression or survival with cerebral blood volume measurements at dynamic susceptibility-weighted contrast-enhanced perfusion MR imaging. *Radiology*.

[44] Hu L. S., Eschbacher J. M., Heiserman J. E. (2012). Reevaluating the imaging definition of tumor progression: perfusion MRI quantifies recurrent glioblastoma tumor fraction, pseudoprogression, and radiation necrosis to predict survival. *Neuro-Oncology*.

[45] Al-Okaili R. N., Krejza J., Wang S., Woo J. H., Melhem E. R. (2006). Advanced MR imaging techniques in the diagnosis of intraaxial brain tumors in adults. *Radiographics*.

[46] Wang Q., Zhang H., Zhang J. (2016). The diagnostic performance of magnetic resonance spectroscopy in differentiating high-from low-grade gliomas: a systematic review and meta-analysis. *European Radiology*.

[47] Choi C., Raisanen J. M., Ganji S. K. (2016). Prospective longitudinal analysis of 2-hydroxyglutarate magnetic resonance spectroscopy identifies broad clinical utility for the management of patients with *IDH*-mutant glioma. *Journal of Clinical Oncology*.

[48] Zhang H., Ma L., Wang Q., Zheng X., Wu C., Xu B. (2014). Role of magnetic resonance spectroscopy for the differentiation of recurrent glioma from radiation necrosis: a systematic review and meta-analysis. *European Journal of Radiology*.

[49] Verhaak R. G., Hoadley K. A., Purdom E. (2010). Integrated genomic analysis identifies clinically relevant subtypes of glioblastoma characterized by abnormalities in *PDGFRA*, *IDH1*, *EGFR*, and *NF1*. *Cancer Cell*.

[50] Prins R. M., Soto H., Konkankit V. (2011). Gene expression profile correlates with T-cell infiltration and relative survival in glioblastoma patients vaccinated with dendritic cell immunotherapy. *Clinical Cancer Research*.

[51] Naeini K. M., Pope W. B., Cloughesy T. F. (2013). Identifying the mesenchymal molecular subtype of glioblastoma using quantitative volumetric analysis of anatomic magnetic resonance images. *Neuro-Oncology*.

[52] Gutman D. A., Cooper L. A., Hwang S. N. (2013). MR imaging predictors of molecular profile and survival: multi-institutional study of the TCGA glioblastoma data set. *Radiology*.

[53] Ellingson B. M., Lai A., Harris R. J. (2013). Probabilistic radiographic atlas of glioblastoma phenotypes. *American Journal of Neuroradiology*.

[54] Carrillo J. A., Lai A., Nghiemphu P. L. (2012). Relationship between tumor enhancement, edema, *IDH1* mutational status, *MGMT* promoter methylation, and survival in glioblastoma. *American Journal of Neuroradiology*.

[55] Tietze A., Choi C., Mickey B. (2017). Noninvasive assessment of isocitrate dehydrogenase mutation status in cerebral gliomas by magnetic resonance spectroscopy in a clinical setting. *Journal of Neurosurgery*.

[56] Leather T., Jenkinson M. D., Das K., Poptani H. (2017). Magnetic resonance spectroscopy for detection of 2-hydroxyglutarate as a biomarker for IDH mutation in gliomas. *Metabolites*.

[57] Ellingson B. M., Cloughesy T. F., Pope W. B. (2012). Anatomic localization of O6-methylguanine DNA methyltransferase (MGMT) promoter methylated and unmethylated tumors: a radiographic study in 358 de novo human glioblastomas. *NeuroImage*.

[58] Hu L. S., Ning S., Eschbacher J. M. (2017). Radiogenomics to characterize regional genetic heterogeneity in glioblastoma. *Neuro-Oncology*.

[59] Barajas R. F., Hodgson J. G., Chang J. S. (2010). Glioblastoma multiforme regional genetic and cellular expression patterns: influence on anatomic and physiologic MR imaging. *Radiology*.

[60] Yang D., Rao G., Martinez J., Veeraraghavan A., Rao A. (2015). Evaluation of tumor-derived MRI-texture features for discrimination of molecular subtypes and prediction of 12-month survival status in glioblastoma. *Medical Physics*.

[61] Lee J., Narang S., Martinez J., Rao G., Rao A. (2015). Spatial habitat features derived from multiparametric magnetic resonance imaging data are associated with molecular subtype and 12-month survival status in glioblastoma multiforme. *PLoS One*.

[62] Macyszyn L., Akbari H., Pisapia J. M. (2016). Imaging patterns predict patient survival and molecular subtype in glioblastoma via machine learning techniques. *Neuro-Oncology*.

[63] Diehn M., Nardini C., Wang D. S. (2008). Identification of noninvasive imaging surrogates for brain tumor gene-expression modules. *Proceedings of the National Academy of Sciences of the United States of America*.

[64] Itakura H., Achrol A. S., Mitchell L. A. (2015). Magnetic resonance image features identify glioblastoma phenotypic subtypes with distinct molecular pathway activities. *Science Translational Medicine*.

[65] Kickingereder P., Götz M., Muschelli J. (2016). Large-scale radiomic profiling of recurrent glioblastoma identifies an imaging predictor for stratifying anti-angiogenic treatment response. *Clinical Cancer Research*.

[66] Grossmann P., Narayan V., Chang K. (2017). Quantitative imaging biomarkers for risk stratification of patients with recurrent glioblastoma treated with bevacizumab. *Neuro-Oncology*.

[67] Delbeke D., Meyerowitz C., Lapidus R. L. (1995). Optimal cutoff levels of F-18 fluorodeoxyglucose uptake in the differentiation of low-grade from high-grade brain tumors with PET. *Radiology*.

[68] Goldman S., Levivier M., Pirotte B. (1996). Regional glucose metabolism and histopathology of gliomas. A study based on positron emission tomography-guided stereotactic biopsy. *Cancer*.

[69] Albert N. L., Weller M., Suchorska B. (2016). Response assessment in neuro-oncology working group and European Association for Neuro-Oncology recommendations for the clinical use of PET imaging in gliomas. *Neuro-Oncology*.

[70] Grosu A. L., Astner S. T., Riedel E. (2011). An interindividual comparison of O-(2-[^18^F]fluoroethyl)-L-tyrosine (FET)- and L-[methyl-^11^C]methionine (MET)-PET in patients with brain gliomas and metastases. *International Journal of Radiation Oncology, Biology, Physics*.

[71] Pauleit D., Floeth F., Hamacher K. (2005). *O*-(2-[^18^F]fluoroethyl)-L-tyrosine PET combined with MRI improves the diagnostic assessment of cerebral gliomas. *Brain*.

[72] Kracht L. W., Miletic H., Busch S. (2004). Delineation of brain tumor extent with [^11^C]L-methionine positron emission tomography: local comparison with stereotactic histopathology. *Clinical Cancer Research*.

[73] Pirotte B., Goldman S., Massager N. (2004). Comparison of ^18^F-FDG and ^11^C-methionine for PET-guided stereotactic brain biopsy of gliomas. *Journal of Nuclear Medicine*.

[74] Rachinger W., Goetz C., Pöpperl G. (2005). Positron emission tomography with O-(2-[^18^F]fluoroethyl)-l-tyrosine versus magnetic resonance imaging in the diagnosis of recurrent gliomas. *Neurosurgery*.

[75] Weber D. C., Zilli T., Buchegger F. (2008). [(18)F]Fluoroethyltyrosine- positron emission tomography-guided radiotherapy for high-grade glioma. *Radiation Oncology*.

[76] Karunanithi S., Sharma P., Kumar A. (2013). Comparative diagnostic accuracy of contrast-enhanced MRI and ^18^F-FDOPA PET-CT in recurrent glioma. *European Radiology*.

[77] Pöpperl G., Götz C., Rachinger W., Gildehaus F. J., Tonn J. C., Tatsch K. (2004). Value of *O*-(2-[^18^F]fluoroethyl)- L-tyrosine PET for the diagnosis of recurrent glioma. *European Journal of Nuclear Medicine and Molecular Imaging*.

[78] Galldiks N., Dunkl V., Stoffels G. (2015). Diagnosis of pseudoprogression in patients with glioblastoma using *O*-(2-[^18^F]fluoroethyl)-L-tyrosine PET. *European Journal of Nuclear Medicine and Molecular Imaging*.

[79] Nihashi T., Dahabreh I. J., Terasawa T. (2013). Diagnostic accuracy of PET for recurrent glioma diagnosis: a meta-analysis. *American Journal of Neuroradiology*.

[80] Suchorska B., Jansen N. L., Linn J. (2015). Biological tumor volume in ^18^FET-PET before radiochemotherapy correlates with survival in GBM. *Neurology*.

[81] Lopci E., Riva M., Olivari L. (2017). Prognostic value of molecular and imaging biomarkers in patients with supratentorial glioma. *European Journal of Nuclear Medicine and Molecular Imaging*.

[82] Bosnyak E., Michelhaugh S. K., Klinger N. V. (2017). Prognostic molecular and imaging biomarkers in primary glioblastoma. *Clinical Nuclear Medicine*.

[83] Spence A. M., Muzi M., Swanson K. R. (2008). Regional hypoxia in glioblastoma multiforme quantified with [^18^F]fluoromisonidazole positron emission tomography before radiotherapy: correlation with time to progression and survival. *Clinical Cancer Research*.

[84] Zagzag D., Zhong H., Scalzitti J. M., Laughner E., Simons J. W., Semenza G. L. (2000). Expression of hypoxia-inducible factor 1*α* in brain tumors: association with angiogenesis, invasion, and progression. *Cancer*.

[85] Evans S. M., Judy K. D., Dunphy I. (2004). Hypoxia is important in the biology and aggression of human glial brain tumors. *Clinical Cancer Research*.

[86] Hoogsteen I. J., Marres H. A., van der Kogel A. J., Kaanders J. H. (2007). The hypoxic tumour microenvironment, patient selection and hypoxia-modifying treatments. *Clinical Oncology*.

[87] Ljungkvist A. S., Bussink J., Kaanders J. H. (2005). Hypoxic cell turnover in different solid tumor lines. *International Journal of Radiation Oncology, Biology, Physics*.

[88] Wack L. J., Mönnich D., van Elmpt W. (2015). Comparison of [18F]-FMISO, [18F]-FAZA and [18F]-HX4 for PET imaging of hypoxia - a simulation study. *Acta Oncologica*.

[89] van Loon J., Janssen M. H., Ollers M. (2010). PET imaging of hypoxia using [^18^F]HX4: a phase I trial. *European Journal of Nuclear Medicine and Molecular Imaging*.

[90] Zegers C. M., Hoebers F. J., van Elmpt W. (2016). Evaluation of tumour hypoxia during radiotherapy using [^18^F]HX4 PET imaging and blood biomarkers in patients with head and neck cancer. *European Journal of Nuclear Medicine and Molecular Imaging*.

[91] Dubois L. J., Lieuwes N. G., Janssen M. H. (2011). Preclinical evaluation and validation of [^18^F]HX4, a promising hypoxia marker for PET imaging. *Proceedings of the National Academy of Sciences of the United States of America*.

[92] Diaz L. A., Bardelli A. (2014). Liquid biopsies: genotyping circulating tumor DNA. *Journal of Clinical Oncology*.

[93] Crowley E., Di Nicolantonio F., Loupakis F., Bardelli A. (2013). Liquid biopsy: monitoring cancer-genetics in the blood. *Nature Reviews Clinical Oncology*.

[94] Kros J. M., Mustafa D. M., Dekker L. J., Sillevis Smitt P. A., Luider T. M., Zheng P. P. (2015). Circulating glioma biomarkers. *Neuro-Oncology*.

[95] Redzic J. S., Ung T. H., Graner M. W. (2014). Glioblastoma extracellular vesicles: reservoirs of potential biomarkers. *Pharmacogenomics and Personalized Medicine*.

[96] Rosenfeld N., Aharonov R., Meiri E. (2008). MicroRNAs accurately identify cancer tissue origin. *Nature Biotechnology*.

[97] Qu S., Guan J., Liu Y. (2015). Identification of microRNAs as novel biomarkers for glioma detection: a meta-analysis based on 11 articles. *Journal of the Neurological Sciences*.

[98] Shi R., Wang P. Y., Li X. Y. (2015). Exosomal levels of miRNA-21 from cerebrospinal fluids associated with poor prognosis and tumor recurrence of glioma patients. *Oncotarget*.

[99] Evans S. M., Putt M., Yang X. Y. (2016). Initial evidence that blood-borne microvesicles are biomarkers for recurrence and survival in newly diagnosed glioblastoma patients. *Journal of Neuro-Oncology*.

[100] Macarthur K. M., Kao G. D., Chandrasekaran S. (2014). Detection of brain tumor cells in the peripheral blood by a telomerase promoter-based assay. *Cancer Research*.

[101] Muller C., Holtschmidt J., Auer M. (2014). Hematogenous dissemination of glioblastoma multiforme. *Science Translational Medicine*.

[102] Francis G., Stein S. (2015). Circulating cell-free tumour DNA in the management of cancer. *International Journal of Molecular Sciences*.

[103] Piccioni D. E., Lanman R. B., Nagy R. J., Talasaz A., Pingle S. C., Kesari S. (2015). Analysis of cell-free circulating tumor DNA in patients with glioblastoma and other primary brain tumors. *Journal of Clinical Oncology*.

[104] Weaver K. D., Grossman S. A., Herman J. G. (2006). Methylated tumor-specific DNA as a plasma biomarker in patients with glioma. *Cancer Investigation*.

[105] Bettegowda C., Sausen M., Leary R. J. (2014). Detection of circulating tumor DNA in early- and late-stage human malignancies. *Science Translational Medicine*.

[106] Diehl F., Schmidt K., Choti M. A. (2008). Circulating mutant DNA to assess tumor dynamics. *Nature Medicine*.

[107] Chen H., Li X., Li W., Zheng H. (2015). miR-130a can predict response to temozolomide in patients with glioblastoma multiforme, independently of O6-methylguanine-DNA methyltransferase. *Journal of Translational Medicine*.

[108] Kushwaha D., Ramakrishnan V., Ng K. (2014). A genome-wide miRNA screen revealed miR-603 as a MGMT-regulating miRNA in glioblastomas. *Oncotarget*.

[109] Verbeek B., Southgate T. D., Gilham D. E., Margison G. P. (2008). *O*
^6^-Methylguanine-DNA methyltransferase inactivation and chemotherapy. *British Medical Bulletin*.

[110] Turcan S., Rohle D., Goenka A. (2012). IDH1 mutation is sufficient to establish the glioma hypermethylator phenotype. *Nature*.

[111] Capper D., Simon M., Langhans C. D. (2012). 2-Hydroxyglutarate concentration in serum from patients with gliomas does not correlate with IDH1/2 mutation status or tumor size. *International Journal of Cancer*.

[112] Husain H., Savage W., Grossman S. A. (2012). Pre- and post-operative plasma glial fibrillary acidic protein levels in patients with newly diagnosed gliomas. *Journal of Neuro-Oncology*.

[113] Todaro L., Christiansen S., Varela M. (2007). Alteration of serum and tumoral neural cell adhesion molecule (NCAM) isoforms in patients with brain tumors. *Journal of Neuro-Oncology*.

[114] Ilhan-Mutlu A., Wagner L., Widhalm G. (2013). Exploratory investigation of eight circulating plasma markers in brain tumor patients. *Neurosurgical Review*.

[115] Hormigo A., Gu B., Karimi S. (2006). YKL-40 and matrix metalloproteinase-9 as potential serum biomarkers for patients with high-grade gliomas. *Clinical Cancer Research*.

[116] Sreekanthreddy P., Srinivasan H., Kumar D. M. (2010). Identification of potential serum biomarkers of glioblastoma: serum osteopontin levels correlate with poor prognosis. *Cancer Epidemiology, Biomarkers & Prevention*.

[117] Albulescu R., Codrici E., Popescu I. D. (2013). Cytokine patterns in brain tumour progression. *Mediators of Inflammation*.

[118] Reynes G., Vila V., Martin M. (2011). Circulating markers of angiogenesis, inflammation, and coagulation in patients with glioblastoma. *Journal of Neuro-Oncology*.

[119] Schneider T., Sailer M., Ansorge S., Firsching R., Reinhold D. (2006). Increased concentrations of transforming growth factor *β*1 and *β*2 in the plasma of patients with glioblastoma. *Journal of Neuro-Oncology*.

[120] Marusyk A., Almendro V., Polyak K. (2012). Intra-tumour heterogeneity: a looking glass for cancer?. *Nature Reviews Cancer*.

[121] Hambardzumyan D., Bergers G. (2015). Glioblastoma: defining tumor niches. *Trends in Cancer*.

[122] Aerts H. J. (2016). The potential of radiomic-based phenotyping in precision medicine: a review. *JAMA Oncology*.

[123] Limkin E. J., Sun R., Dercle L. (2017). Promises and challenges for the implementation of computational medical imaging (radiomics) in oncology. *Annals of Oncology*.

